# Involving deprived communities in improving the quality of primary care services: does participatory action research work?

**DOI:** 10.1186/1472-6963-7-88

**Published:** 2007-06-18

**Authors:** Peter G Cawston, Stewart W Mercer, Rosaline S Barbour

**Affiliations:** 1 Drumchapel Health Centre, 80-90 Kinfauns Drive, Glasgow, G15 7TS, UK; 2 Department of General Practice and Primary Care, Division of Community-based Sciences, University of Glasgow, 1 Horselethill Road, Glasgow, G12 9LX, UK; 3 School of Nursing and Midwifery, University of Dundee, 11 Airlie Place, Dundee, DD1 4HJ, UK

## Abstract

**Background:**

Participation by communities in improving the quality of health services has become a feature of government policy in the United Kingdom. The aim of the study was to involve a deprived community in the UK in shaping quality improvements of local primary care services. The specific objectives were firstly to create participation by local people in evaluating the primary care services available in the area and secondly to bring about change as a result of this process.

**Methods:**

The methods of participatory action research was used. The study was set in an area of high socio-economic deprivation served by a 'Local Health Care Co-operative' in a peripheral housing estate in Glasgow, Scotland. 72 local residents took part in 11 focus groups: eight of these were with community groups and three with other residents. 372 local residents completed questionnaires either by brief face-to-face interviews (114) or by self or carer completion (258).

**Results:**

The study group produced recommendations on physical access to the health centre, time constraints in accessing services and problems encountered in individual relationships with health staff. They also highlighted the social gap between health service providers and the daily life of community residents. Action was taken to bring these recommendations to the attention of the Primary Care Organisation.

**Conclusion:**

Participatory action research was used to involve a deprived community in the UK in a 'bottom-up' approach aimed at improving quality of local primary care services. Although successful in creating a partnership between academic researchers and lay researchers and participation by local people in evaluating the primary care services available in the area, the impact of the study in terms of immediate action taken over specific issues has been modest. The possible reasons for this are discussed.

## Background

Participation by communities in improving the quality of health services has become a feature of government policy in the United Kingdom. However, there has been relatively little evaluation of this activity, particularly when compared to the speed and extent of the recent changes that have been introduced in the NHS. Case studies have suggested that modest changes in quality may result from community participation [1].

Participatory action research is an approach to research [2,3] which emphasises the political nature of how knowledge is created. Research participants are seen not as the passive objects of research knowledge but as active contributors to shaping knowledge about their world and the problems they face. Participatory action research has been used principally in health development among marginalised indigenous communities in the 'Two-Thirds World' but could be used more widely in quality improvement in health services [4], for example as a method of engagement with those who do not traditionally take part in 'lay involvement' activities, such as socio-economically disadvantaged communities in the industrialised world.

We describe a study that draws on participatory action research theory to involve a deprived community in the UK in improving the quality of local primary care services. The purpose of the study was firstly to create participation by local people in evaluating the primary care services available in the area and secondly to bring about change as a result of this process. In this paper we discuss the usefulness of the participatory action research approach in achieving these two aims.

## Methods

### Theoretical considerations

A central tenet of participatory action research is that the subjects of research should participate in all stages of the process of knowledge formation [2,5]. This includes setting the questions to be asked, determining how information will be sought, shaping how information is interpreted and taking part in activities to bring about change. This has two implications for the research process. Firstly it blurs the boundary between expert and non-expert by de-professionalizing the research process, as the two groups becoming co-learners. Secondly it gives central place to a concern for participation and change. The experiences, attitudes and beliefs of those taking part are not conceived of as objective 'things'. The research process develops an understanding of how subjective worlds are expressed *in order to *bring about changes desired by the subjects of research. This has been described as favouring 'learning' rather than 'proving' in a traditional scientific sense [6].

The validity (or truth value) of research from this perspective is derived firstly from the extent to which non-expert participants shape the project (authenticity of participation) and secondly from the extent to which the project and its findings create the conditions for action (usefulness). These are given primacy over other considerations such as the 'correctness' of research procedures and the reproducibility of findings, favouring multiple voices (dialogue) over a single authoritative voice (the academic monologue).

### Research setting

The study was set in the locality served by a 'Local Health Care Co-operative', a small primary care organisation (PCO) in a peripheral housing estate in Glasgow, Scotland. The area covers a population of 22,700 people who are recorded as having high levels of socio-economic deprivation. 53% of people registered with the PCO are classified as being in the poorest deprivation category. Standardised mortality in the area is considerably higher than the national average.

### Producing a project

A small research group made up of four local residents and an academic general practitioner met regularly over a period of one year to plan and implement the study. The academic member of the group – who undertook the project as part of a masters degree in primary care – kept careful field notes of this process and was supported by a senior non-clinical research supervisor with expertise in qualitative methods. The study was granted ethical approval by the Local Research Ethics Committee. The lay members of the research group comprised a lay health worker who had experience of campaigning on local issues, and three local residents who were recruited on the basis that they had expressed an interest in the study. Two of these had previous volunteer involvement with local health projects. The academic member of the group also works in the locality as a part-time general practitioner, but doesn't live in the area.

### Producing data

The group chose two methods to gather data in order to inform action: a questionnaire survey and focus groups. The questionnaire was used on the basis that it would allow flexibility and would be accessible to the non-professional members of the group in both carrying out the survey and analysing its findings. The aim of the questionnaire was to give access to a wide range of brief replies so as to allow the research group to recreate a sense of the breadth and multiplicity of issues and perspectives. The group developed and piloted a set of open questions that could be used flexibly in a variety of settings. These open questions were:

1. What have you liked about the services which you've used?

2. What have you disliked about the services which you've used?

3. How do you think we could improve our services?

4. Are there any other health services which you think we should be providing?

5. What do you think would most improve the lives of people living in your local community?

These five items were used to conduct brief one to one face-to-face interviews, lasting approximately five minutes. In addition a survey tool using the same questions was distributed locally for self-completion either individually or with the help of carers. All replies were hand-written and then transcribed and collated.

Focus groups were set up to permit a more in-depth exploration of issues [7,8] and to give those taking part a greater sense of personal contact with the project, possibly encouraging them to become personally involved with taking action with the issues raised in the groups. A similar set of questions was used as the topic guide for semi-structured focus group discussions. These were:

1. What's been a good experience of getting help with health problems?

2. What's been a bad experience?

3. What would you like to have been different?

4. How do you think the health services which you receive could be improved?

5. What sorts of things do you think would most improve the lives of people living in your local community?

The academic general practitioner and one of the local residents in the study group facilitated. Discussion was provoked by reference to a popular television medical drama. Similar questions to those used in the questionnaire provided a structure, but participants were encouraged to explore the issues in greater depth using personal experiences and by entering into discussion with others in the focus group. They were asked to evaluate their experiences, develop criticisms and explore what would be a good service. Discussions were audio-recorded and transcribed verbatim.

### Producing a community

The research group did not aim to provide a comprehensive picture of 'needs assessment' through a formally identified sample of a definable community. The project was built on the view that community is an ambiguous and complex concept [9]. The aim of the research group was to produce a sense of the multiplicity of voices in the local area and at the same time to create momentum for bringing about change within the local health services on issues that were raised by people taking part. In keeping with this aim, the picture of community was taken using the knowledge provided by the local residents in the research group.

The four local residents in the group played a central role therefore in deciding who should be invited to contribute to the questionnaires and focus groups. This was initially carried out with community groups with whom the research group had some pre-existing contact. This was extended to members of groups with whom they had no previous contact but who they felt had not yet been heard; to clients in contact with local health and social services and to individuals in public spaces such as the health centre, shopping centre and bingo hall.

**Figure 1:**
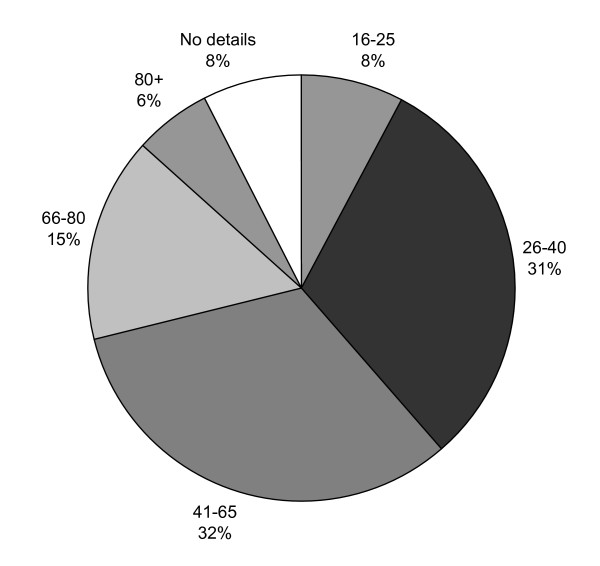
Age of questionnaire respondents (in years).

372 questionnaires were completed either by brief face-to-face interviews (114) or by self or carer completion (258). Of those who indicated their gender 71.5% (n = 340) were women. Ages ranged from 16 to over 80 (Figure 1). Questionnaires were completed in a variety of different settings (Table 1).

**Table 1:** Drumchapel Health Focus Questionnaire Respondents

*Community Groups:*	Responses
Drumchapel Community Health Project	6
Drumchapel Disabled Action Group	25
Focal Point Day Centre for the Elderly	26
Men's Group	3
Drumchapel Family Learning Centre	12
Caring Over People's Emotions	19
Princess Royal Trust for Carers	15
Drumchapel Opportunities	28
Drumchapel Home Visiting Scheme	24
Misc.	3
*Drumchapel Health Centre:*	
Arndale Resource Centre	15
Child Health Clinics	10
GP Surgeries	120
District Nurses	9
*Public Areas*	
Bingo	20
Shopping Centre	37
**TOTAL:**	**372**

Focus group participants were recruited through community groups or by contacting people who had expressed an interest when completing the questionnaire. 72 people attended 11 focus groups, of whom 61.1% (n = 72) were women. Ages ranged from under 16 to over 80. Eight focus groups were held with a variety of community groups and three in a community centre with local residents (Table 2).

**Table 2:** List of Focus Groups Held

**Focus Groups**	**Number attending**
Community Health Project (health volunteers)*	7
Antonine Court (disabled)*	7
Focal Point (elderly)*	9
COPE (mental health)*	6
Family Learning Centre/Child Health Clinics (parents)*	6
Men's Group (alcohol problems)*	9
Community centre (1)	6
Community centre (2)	4
Community centre (3)	5
Essenside Youth Group (younger teenagers)*	5
G15 Project (older teenagers)*	8
	
*Community groups	
**Total**	**72**

### Producing findings

The questionnaire replies were used to generate a spread of responses. The research group read transcripts of all the replies. Each reply was placed into preliminary categories, which were grouped into themes. The group contrasted different meanings that could be given within the themes and explored the relationship between them. The lay members of the group were encouraged to use personal experiences or those of relatives and friends when these were felt to be relevant to the data. This allowed them to draw on their own local knowledge to enrich the themes derived from the data and to provide unexpected insights into the questionnaire replies. The research group processes provided a form of second order analysis of the broad spread of views expressed through the questionnaire.

The themes provided by these discussions were used in two ways. Firstly in a semi-quantitative way to identify issues most frequently discussed, and secondly to provide a basis for discussion of issues emerging within each theme, focusing in particular on possible changes that were demanded of local health services.

In order to provide a further dimension to the issues emerging from this discussion of the questionnaire replies, the themes were used as the basis for analysis of the focus group transcripts. One person (the academic practitioner) identified and coded examples that supported or contradicted the ideas developed by the research group. NUD*IST software was used to facilitate this purpose. Issues were framed or given greater complexity to take into account contrasting views developed in the focus groups, thus providing a 'third order' analysis of the findings.

### Producing action

**Figure 2:**
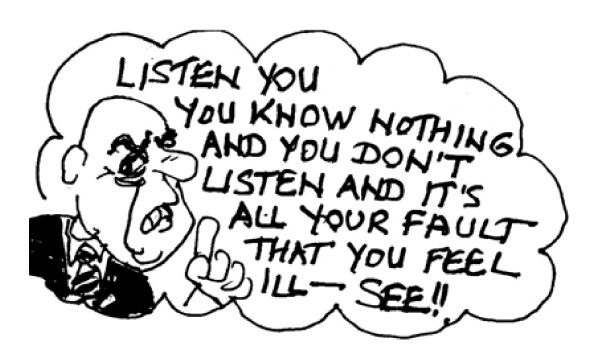
'Respect' (produced by a focus group participant).

The primary means for generating a response to the issues identified was by local dissemination of the findings. This was carried out during the course of the study by means of regular newsletters. At the end of the study detailed reports were published with specific recommendations. Written publications were given added weight by the use of direct quotes, photographs and cartoons (Figure 2) drawn by a local artist who took part in one of the focus groups. The newsletters were sent to people who had taken part in the study as well as to local health and social services. The full report was sent to selected local health care practitioners and to the primary care organisation executive.

During the period of the study observational field-notes and documentary records were kept. The academic practitioner has continued as a participant in the primary care organisation and was able to observe actions that have taken place subsequent to the end of the project.

## Results

### Access: physical accessibility

#### Finding

The research group identified physical access as a prominent issue with parents of young children and with a disabled action group. On the basis of this, three health centre users with physical impairments were asked to inspect the health centre. A list of issues was developed and a report sent to the Chairman of the city Access Panel for advice for comment before being circulated to the primary care organisation.

#### Action

As an overt response to this report the primary care organisation invested in automatic doors, adjustable examination couches and minor structural changes to the local health centre building. The doors were ceremonially 'opened' by one of the local people who had carried out the inspection.

### Access: time constraints

#### Finding

The theme of access to health care covered a wide range of issues relating to time constraints in the health service, including waiting times for appointments, late running surgeries, short consultation times, unpredictability of appointment availability, and limited opening hours. There were many reasons why issues of time were considered as being important. These included difficulty in articulating urgent concerns, anxiety about diagnosis or treatment, being deterred by crowded waiting rooms and feeling unable to take time off work to consult.

#### Quote 1

*'You get stressed out with worrying and stress can bring out all sorts in you, I've suffered stress and it's brought out depression and anxiety and it's no fun, the waiting time's a killer*...

*If you're not well if you're going to have to wait a week or something, you're gonna get worse and worse.' *[Focus Group H]

#### Action

As part of a wider development planning process, an access improvement group was set up by the PCO. A number of changes have since been made such as 'open access' to some allied health professional services and 'advanced access' to general practice. While the project findings were referred to in the PCO plans, the changes that have resulted reflect national and health board initiatives. More local issues such as the availability of services out of normal working hours and difficulties faced by teenagers in accessing emergency contraception were not acted upon.

### Engagement: individual relationships

#### Finding

The research group findings emphasised the centrality of interpersonal relationships with health staff. This included themes of approachability, genuine relationships, and respect. In the context of multiple barriers to accessing health care, the 'approachability' of health care providers was seen as having a powerful influence on the ease with which patients felt they could approach and talk to them.

#### Action

With explicit reference to the findings of the project, the PCO set up a 'patient-centredness' group with the remit of reflecting the importance of relationships between patients and staff in the organisation's development planning process. Plans were partially implemented for example by providing protected learning time for multi-disciplinary communication skills training.

### Engagement: community relationships

#### Findings

The fact that none of the GPs working in the health centre live or socialise in the area was contrasted with GPs on television programmes who lived near to and socialised with their patients. Social distance added to system barriers to access.

As a result of 'social distance', it was felt that health staff could not really understand "the sort of life that people in Drumchapel are actually living". Health advice and the configuration of services seemed to fail to take into account the realities of daily life such as limited local shopping facilities, public transport issues, difficult choices on low incomes, the effects of living with damp housing, conflicting family demands, fear of violence, boredom or social isolation.

#### Quote 2

*'She is not used to dealing with folk on this low income that has to balance one thing against another, the way that she was talking you get the impression that I was spending all my money on cakes. I said listen you feed two people off of thirty pounds and I don't just mean feed, I need to buy soap powder, disinfectant, bleach, shampoo, toilet rolls, this all comes off my money and you tell me how you could buy bags and bags of chocolate biscuits off that because I certainly can't and I consider myself a good shopper. It came to the point that I felt she's either patronising me or putting me down, but by the end of this interview, if she had come up with an idea that had made me eight stone by Friday, I was so up and against her that I wasn't listening and it was like a competition and (she said) "I just do this and I just do that"... If I had her income I could do what she could do!' *[Focus Group D]

#### Action

Shortly after the completion of the project the PCO took the decision to fund a community health action team whose purpose would be to provide a bridge between health services and community and social projects. The report of the research group appears to have played a part in supporting this decision. The team has built on a previous community health project and has engaged lay workers and volunteers. One of the lay members of the research group obtained employment with the action team. The community health action team continues to undertake projects and represents the PCO on community bodies and provides lay members to the PCO executive group.

## Discussion

The purpose of the study was to create participation by local people in evaluating their primary care services and to bring about change as a result of this process. Although successful in creating participation, the impact of the study in terms was relatively modest.

### Strengths and weaknesses

The choice of methods reflected the needs of participatory design and analysis. Key requirements were simplicity and flexibility. The study utilised the lay group member's own local understanding of who represented their community. This had the strength of building on existing contacts and networks. This strength however also limited the data since certain sectors of the community (for example men) were under represented.

The questionnaire developed by the group was not a standardised instrument. The accessible format of the survey data allowed lay and academic members of the research group to develop a shared understanding of concepts and themes. The deliberate use of subjective interpretations and experiences in interpreting responses to the questionnaires had the potential to undermine the extent to which the findings accurately represented those responses. However, the process gave access to local knowledge and interpretations that may have been inaccessible to an academic researcher alone. However, the methods used in the study are comparable with methods used in evaluating whole system approaches to health care development [10] and our use of 'mixed methods' is justified by evidence-based systematic reviews of patient participation techniques [11].

In terms of the possible transferability of the study approach, the importance of the role of the academic clinician (and the indirect role of his research supervisor) in supporting the process of the project and working with the local research team should also not be, in our view, underestimated in terms of the 'success' of the project. The fact that the clinical academic was also a local GP, and perhaps served as a bridge between 'town and 'gown, may also have been an important element. Additionally, the locality in which the project took part has a strong tradition of community care and community development, and the population who participated may therefore have been more receptive to community-based projects than other areas of high deprivation elsewhere.

The purpose of the study was to create participation and bring about change. As an application of participatory action research theory the study provides a contribution to areas of debate that are pertinent to health care organisations seeking to obtain the views of the community in developing services. A key question for the research group was how to build a picture (to re-present) the local community. Community can mean many things however [12]. It can be seen as a unit (e.g. defined by a locality) or as a plurality of units, derived from external markers (e.g. gender, age, race), from activities and relationships (e.g. membership of groups) or from subjective positions (e.g. acceptance or rejection of community membership). Community can also be understood as a cultural ideal or "folk model" that contrasts with the existential reality of isolation and fragmentation [13].

As a result the community represented by the research group is of people who participate in community groups, use services and enter public spaces that are traditionally associated with women (supermarket, bingo etc). This is clearly not the only community served by the primary care organisation. It is however a significant community or communities that may not have been accessed using standard statistical or purposive sampling methods. This is not to argue that the latter are unnecessary, but rather to urge caution in accepting studies that use such methods as the sole legitimate means to represent a community. The use of a participatory action research approach allowed the representation of a lay construct of community that relied neither on the opinions of a vocal few nor on the expertise of an external observer.

#### How 'authentic' is participation?

The study adopted the perspective that authentic participation depends on full involvement in shaping and creating knowledge [2]. Local people were therefore involved in planning, implementing and analysing the research project. Traditionally participation has been represented in terms of a ladder that spans coercion to full control of decision-making [2,9]. This model does not however raise into question the processes by which knowledge itself is formed [4,14]. The knowledge on which decisions are taken already determines the rules and the direction of those decisions. From this perspective knowledge is not neutral but reflects the values of those who have control of the production of knowledge. Authentic participation therefore demands early involvement in setting the questions and developing the answers to inform how decisions are formulated [9].

### Meaning and implications for policy and practice

#### Does participation lead to change?

At a superficial level it could be demonstrated that the primary care organisation acting on a number of recommendations given by the research project. The participation of the lay members of the research group raised the profile of lay involvement within the primary care organisation and the existence of research findings provided legitimacy for their ongoing contributions within the PCO in new roles as lay workers and lay representatives. The participatory nature of the study, the use of direct quotes and illustrations, gave a 'real' quality to the findings that was generally accepted when presented to health service personnel. Physical changes made within the health centre symbolised a practical response to specific recommendations.

However responses to the recommendations were limited and many recommendations were not acted on even when these only had small resource implications. For example, on this problem of physical access, the ease of response was facilitated by the fact that primary care services in the area are concentrated in one relatively new building that had basic existing levels of accessibility such that major investment and re-building was not required. In addition, where action was taken, this action tended to reflect national and wider priorities rather than locally identified issues.

Responses to the recommendations relied on the good will of allies within the health service rather than carrying direct influence in their own right. This required the existence of 'champions' within the existing structures to take up issues, and made any changes vulnerable to the other competing demands on management or the movement of personnel as a result of health services restructuring. Despite a strong commitment to the project by the primary care organisation's executive members, the influence resulting from the process became part of a wider diffuse network, competing with a plurality of influences and structural constraints, many of these outside the limited control of management at a local level [15]. The 'lay' perspective may have become more prominent as a feature of the "organisational landscape" [16] but the responsibility for decisions remained outside their control.

The lack of significant influence may have related to the dissemination methods of findings (ie., newsletters to the GPs and LHCC). More active methods could have been used involving more direct advocacy work by the lay research team and by using more local healthcare professionals as key informants, as described by the project of Murray and colleagues [17]. The current project also did not directly explore the views of healthcare staff about changes in their own work organisation, even though the study took place at a time when a forum was being created for shared decision making (i.e., the Local Health Care Cooperative or LHCC). However, it was the LHCC that commissioned the project and provided funding and logistic support (shared with the university department). The study therefore evolved within local health services and involved local medical and nursing staff in its development and in the local dissemination of findings.

Factors that might explain its limited impact at a local level might be:

(1) Its broad scope addressed issues of interest to the participants (the community) but these did not directly mesh with the concerns of specific organizational work units in the local health services (who were uncertain, for example, how to respond to concerns about lack of respect for certain groups of patients)

(2) The limited resources available to the LHCC and the as yet immature connections with both local work units and the wider health service (spending a fixed sum of money on new doors was a typical small investment of which the LHCC was capable at the time, without being able to bring larger influence to bear)

(3) It took place at an early stage in the process of organizational change, when this was being actively resisted by some. It may have been perceived by some staff to be 'external' as it was associated with moves to a wider social focus away from traditional professional led practice

(4) Continual reorganization meant instability in terms of personnel (e.g. supportive middle managers) and also major structural reorganization subsequent to the project meant that processes in place to try and take further action were lost.

The lack of influence found in this study however also resonates with Harrison's analysis that communities are precluded from exercising meaningful control within the current structuring of health services [15,16]. An extensive review of people's participation over three decades concluded that even the most firmly established gains of organised groups occupied a precarious position and could be obliterated by changes in the wider political sphere [18]. Previous work in Scotland has also reported that national top-down priorities also swamped local issues [19]. Efforts such as the one described here exist not in a vacuum but in a complex network of power relationships that preclude any simplistic conclusion that community participation results in community empowerment [20,21].

'Social distance' between doctors and the local community alos emerged as an important issue, and we have reported on this in a different paper [22]. Social distance was highlighted in a number of ways:

(1) Lack of understanding of the environmental, financial, family and social concerns of patients, and how these affected how people cope with illness

(2) Badges of social distance such as accent, clothes, appointment systems, layout of consulting rooms,

(3) Gaps in forms of communication, language used

(4) Social class division, i.e. almost no professional staff resident locally, no social contacts outside work setting, badges of wealth (e.g. car)

(5) Lack of respect, prejudice, labelling of patients based on social group features

The implications are that patients living in deprived areas have extra barriers to overcome in accessing formal health services. They are also cut off from informal education and advice networks with health professionals (e.g. being able to approach them informally for guidance as neighbours, sports partners, through school, club membership etc). We feel this is an important area for future research.

## Conclusion

Participatory action research was used to involve a deprived community in the UK in a 'bottom-up' approach aimed at improving quality of local primary care services. Although successful in creating a partnership between academic researchers and lay researchers and in creating participation by local people in evaluating the primary care services available in the area, the impact of the study in terms of immediate action taken over specific issues has been modest.

## Competing interests

The author(s) declare that they have no competing interests.

## Authors' contributions

PGC conceived and designed the study, helped collect data, and carried out the analysis and interpretation of the data as part of a MSc thesis. He also wrote the first draft of the article, and gave final approval to the final draft. RSB helped in the design of the study, and in the preliminary interpretation of the data. She also helped revised the first draft giving critical intellectual input. She also gave final approval. SWM discussed the conception and design at an early stage with PC. He helped in the analysis and interpretation of the data, revised several versions of the manuscript, and also gave critical intellectual input into this process. He produced the final draft which he approves.

All authors read and approved the final version of the manuscript.

## Pre-publication history

The pre-publication history for this paper can be accessed here:



## References

[B1] Crawford MJ, Rutter D, Manley C, Weaver T, Bhui K, Fulop N, Tyrer P (2002). Systematic review of involving patients in the planning and development of health care. BMJ.

[B2] Rifkin S, Lewando-Hunt G, Draper A, Rifkin S, Lewando-Hunt G, Draper A (2000). Theoretical constructs of Community Participation. Participatory approaches in health promotion and health planning: A literature review.

[B3] Macaulay AC, Commanda LE, Freeman WL, Gibson N, McCabe ML, Robbins CM, Twohig PL (1999). Participatory research maximises community and lay involvement. North American Primary Care Research Group. BMJ.

[B4] Cawston PG, Barbour RS (2003). Clients or citizens? Some considerations for primary care organisations. BJGP.

[B5] Meyer J (2000). Using qualitative methods in health related action research. BMJ.

[B6] Morrison B, Lilford R (2001). How can Action Research Apply to Health Services?. Qualitative Health Research.

[B7] Barbour R (1995). Using focus groups in general practice research. Family Practice.

[B8] Kitzinger J, Barbour RS, Barbour RS, Kitzinger J (1999). The challenge and promise of focus groups. Developing Focus Group Research: Politics, Theory and Practice.

[B9] Rifkin SB, Draper A, Hawdon D (2000). Major issues arising from the literature review on participatory approaches to health improvement. Improving health through community participation.

[B10] Jee M, Popay J, Everitt A, Eversley J (1999). Evaluating a whole systems approach to primary care development.

[B11] Ryan M (2001). Eliciting public preferences for health care: a systematic review of techniques. Health Technology Assessment.

[B12] Cohen AP (1985). The symbolic construction of community.

[B13] Jewkes R, Murcott A (1996). Meanings of Community. Social Science and Medicine.

[B14] Freire P (1996). Pedagogy of the Oppressed.

[B15] Harrison S, Ahmad W (2000). Medical Autonomy and the UK State 1975 to 2025. Sociology.

[B16] Harrison S, Mort M (1998). Which Champions, Which People? Public and User Involvement in Health Care as a Technology of Legitimation. Social Policy and Administration.

[B17] Murray SA, Tapson J, Turnbull L, McCallum J, Little A (1994). Listening to local voices: adapting a rapid appraisal technique to assess health and social needs in a general practice. BMJ.

[B18] Stiefel M, Wolfe M (1994). A voice for the excluded: popular participation in development.

[B19] Brown C, Lloyd S, Murray S (2006). Using consecutive rapid participatory appraisal studies to assess, facilitate, and evaluate health and social change in community settings. BMC Public Health.

[B20] Prior D, Stewart J, Walsh K (1995). Citizenship: rights, community and participation.

[B21] Hogg C (1999). Patients, power & politics: from patients to citizens.

[B22] Mercer SW, Cawston P, Bikker AP (2007). Quality in general practice consultations; a qualitative study of the views of patients living in an area of high socio-economic deprivation in Scotland. BMC Family Practice.

